# The Genetic Background of Central Serous Chorioretinopathy: A Review on Central Serous Chorioretinopathy Genes

**DOI:** 10.7150/jgen.55545

**Published:** 2021-01-01

**Authors:** Konstantinos Giannopoulos, Maria Gazouli, Klio Chatzistefanou, Anthi Bakouli, Marilita M Moschos

**Affiliations:** 1 First Department of Ophthalmology, Gennimatas General Hospital, National and Kapodistrian University of Athens, School of Medicine, Athens, Greece; 2 Department of Ophthalmology, General Hospital of Sitia, Sitia, Greece; 3 Department of Basic Medical Sciences, Laboratory of Biology, National and Kapodistrian University of Athens, School of Medicine, Athens, Greece; 4 Department of Ophthalmology, Elpis General Hospital, Athens, Greece

**Keywords:** Central Serous Chorioretinopathy, Diagnosis, Genes, Pathophysiology, Review

## Abstract

Central serous chorioretinopathy is characterized by neurosensory detachment of the central retina secondary to fluid leakage through the retinal pigment epithelium. Though it has an incidence of 9,9 per 100.000 in men and 1,7 per 100.000 in women, it is the fourth most common retinal disorder. Central serous chorioretinopathy patients present with blurred vision, central scotoma, metamorphopsia, micropsia and mild color discrimination. It is usually a self-limited disorder with nearly none or minimal visual impairment but in some patients the disease persists and may cause severe visual impairment. Central serous chorioretinopathy pathophysiology is not well understood. Choroid, retinal pigment epithelium and hormonal pathways seem to play important roles in central serous chorioretinopathy pathophysiology. Also, familial cases of the disease indicate that there is a genetic background. The identification of certain disease genes could lead to the development of better diagnostic and therapeutic approaches for central serous chorioretinopathy patients.

## Introduction

Central serous chorioretinopathy (CSCR) is the fourth most common retinal disorder 1. It is characterized by neurosensory detachment of the central retina secondary to fluid leakage through the Retinal Pigment Epithelium (RPE) 2, 3, 4.

CSCR was first described by Von Grafee in 1866^2^, ^5^.Almost 100 years later Maumamme was the first who described the angiographic characteristics of the disease 2, 5. A few years later Gass described in more details the angiographic features of the disease and named it as central serous choroidopathy 6, 7, 8.

In a population-based retrospective cohort and case control study in Olmed county in Minessota, Kitzmann et al found that CSCR has an incidence of 9,9 per 100.000 in men and 1,7 per 100.000 in women. Men are affected, approximately, six times higher than women. CSCR affects predominantly middle aged people (30-50 years old) with a mean age of onset around 40 years old (39-41 years) 9. Asians have been reported to have higher incidence of CSCR than Caucasians 9, 10.

CSCR is a disorder of unknown etiology. Several risk factors for the disease have been reported 3, 4, 5. Corticosteroids usage is widely accepted as a strong risk factor for CSCR 3, 5, 11, 12. Other main risk factors are endogenous Cushing syndrome, pregnancy and type A personality 3, 5, 13.

Familial cases of CSCR indicate that there is a genetic predisposition to the disease 3, 4, 5, 14, 15, 16.

## Clinical features and diagnosis

CSCR, usually, is a self-limited disorder that resolves within 3-4 months with nearly none or minimal visual impairment (acute form) but in some patients subretinal fluid and neurosensory retinal detachment persists for more than 3 to 4 months (chronic form) 4, 5, 17, 18. The duration of the disease to be characterized chronic is set arbitrarily between 3-6 months but most recent papers define chronicity after 3 months of persistent fluid 4, 5, 17, 18. Chronic CSCR may cause severe visual impairment due to permanent damage to photoreceptors and Retinal RPE. Also, choroidal neovascularization is much more likely to develop with chronic CSCR 3, 4, 5, 19.

Patients with CSCR presents with blurred vision, central scotoma, metamorphopsia micropsia and mild color discrimination. Presenting visual acquity is usually between 20/20 to 20/80. CSCR patients presents, usually, with unilateral symptoms but evidence of subclinical disease in the other eye may be found in a significant proportion of patients (30-40%). Fundoscopy reveals round or oval localized detachment of the neurosensory retina. Subretinal blood or lipid exudates are absent. RPE irregularities and atrophy may be noticed, mostly indicating chronicity or previous episodes. Amsler test is pathologic showing relative scotoma and distortion of straight lines 5, 20, 21.

CSCR diagnosis is often straightforward but a number of clinical entities produce serous neurosensory detachment and should be differentiated from CSCR. The main diseases that may mimic CSCR are: age-related macular degeneration, polypoidal choroidal vasculopathy, choroidal tumors, optic disc pit, dome-shaped macula and inflammatory conditions like Vogt-Koyanagi-Harada syndrome and posterior uveitis 21, 22. Apart from a meticulous clinical examination including fundoscopy, multimodal imaging using Optical Coherence Tomograpgy (OCT), Fluorescein Angiography (FA), Indocyanine Green Angiography (ICGA) and fundus autofluorescence are used to differentiate CSCR from other diseases 4, 5, 21, 22.

## Pathophysiology

Pathophysiology of CSCR is, still, not well understood. Choroid, RPE and hormonal pathways seem to play important roles in CSCR pathophysiology. Also, numerous reports of familial CSCR indicate that there is genetic predisposition to CSCR 3, 4, 5, 14, 15, 16.

Currently, choroid seems to play the most important role in the pathogenesis of CSCR. RPE dysfunction is also important in CSCR pathophysiology but the presence of choroidal staining on ICGA shows that choroidal dysfunction is the main contributing factor 2, 4, 5. Guyer et al, using indocyanine green videoangiography, showed diffuse choroidal hyperpermeability from active leakage sites that were not seen with FA. RPE detachments occur as a result of choroidal hyperpermeability 2, 23. RPE detachments may rip or decompensate and RPE leakage causes neurosensory retinal detachment 3, 23. Further evidence for the primary role of the choroid comes from a number of studies that showed choroidal ischemia as well as choroidal leakage in patients with CSCR 24, 25, 26, 27, 28.

Areas of RPE leakage in FA are usually contiguous with areas of choroidal hyperpermability so it is a rational hypothesis that RPE alterations are secondary to choroidal abnormalities 3, 5. RPE leaks are not universally present in all areas of choroidal staining suggesting that RPE may overwhelm the stress from choroidal pathology 3, 5, 26, 28, 29. Therefore the interaction between the two layers (Choroid and RPE) plays a crucial role in CSCR pathophysiology 2, 4.

Tittl et al showed that foveal fundus pulsation amplitude was significantly higher in CSCR affected eyes than in control eyes providing evidence of abnormal choroidal perfusion 4, 30. Also, in another study, Tittl et al showed impaired foveal choroidal blood flow regulation in patients with inactive CSCR suggesting that in CSCR patients there is a persistent abnormal autoregulation in the choroidal blood flow 2, 31.

More supporting evidence for the primary role of the choroid comes from studies using enhanced depth imaging optical coherence tomography (EDI-OCT) that found thickened choroid in both eyes of CSCR patients 3, 5, 32, 33, 34. CSCR belongs to the pachychoroid disease group. This group includes several diseases that have common features like increase choroidal thickness (diffuse or focal) and hyperpermeability of the choroid on ICGA 35.

There is strong evidence that corticosteroids both exogenous and endogenous contributes to the development of CSCR. Also, male gender, stress and pregnancy have been associated as risk factors for CSCR. Complex hormonal interactions including the hypothalamus-pituitary-adrenal axis, the autonomic nervous system and cellular hormonal receptors suggest possible explanations for the above associations 2, 3, 4, 5.Preclinical studies showed that overactivation of the mineralacorticoid pathway increased choroidal thickness and produced RPE alterations and subretinal fluid like CSCR 4, 36.

## Purpose-methods

A literature review in PUBMED, using various keywords, regarding the genes that have been implicated in the pathogenesis of CSCR was performed.

## The Genetic background of Central Serous Chorioretinopathy

In a pivotal study, Weenink et al examined 80 relatives, mainly siblings, of 27 chronic CSCR patients. They found chronic CSCR to be familial in 52% of the families. In 14 out of 27 families two or more members had retinal lesions of chronic CSCR or multiple areas of RPE atrophy. In general 35 out of the 80 relatives that participate in the study were affected. Since the study was mainly restricted to one generation, researchers were not able to make any conclusion regarding the mode of inheritance 14.

In a recent study, 103 subjects from 23 families were phenotyped by Van dijk et al. 39 out of 103 subjects were previously diagnosed with CSCR and 64 were undiagnosed family members. Of the 64 undiagnosed family members six (9%) were diagnosed with CSCR and 27(42%) showed RPE changes suggestive of CSCR on multimodal imaging. Therefore, 44 %( 45 out of 103) of the 103 included subjects in the study were diagnosed with CSCR, 26 %( 27 out of 103) had a CSCR suspicion and 30 %( 31 out of 103) had no signs of CSCR. Generally, 52% of family members of patients in this study were detected with (suggestive) CSCR 15.

The same group of researchers performed exome sequencing on 72 individuals of 18 families in which multiple members were diagnosed with chronic CSCR. Participants of the study were divided in three groups. First group (33 subjects) those affected with CSCR, second group (18 subjects) those suggestive of CSCR and third group (21 subjects) those unaffected. Segregation analysis was performed for all 18 families. Variants which were present in the affected individuals and absent in unaffected individuals were retained. Variants that segregated in at least two families were retained for further evaluation. Totally, 11 rare genetic variants from the following genes PTPRB, SETD2, PWP1, ABCA9, AT2B2, ZFAND4, MROH5, ZAN, SHISA6, DCP1A and PPM1E were found to segregate in two families. Apart from ZAN gene all the others are expressed in the retina and RPE. In addition, they observed in 28 genes two or more different heterozygous genetic variants that segregated in two or more families. However, none of the genes showed consistent associations in both the gene burden analysis in the case-control cohort and in the family gene burden analysis. Researchers concluded that in familial CSCR a mendelian inheritance of variants in one or a limited number of genes could be excluded. Instead they stated that familial CSCR may be a heterogeneous mendelian disease which is caused by variants in many different genes or alternatively CSCR is a complex disease that both genetics and environmental factors contribute to its development 16.

## Complement factor H

Complement factor H (CFH) plays an important role as an inhibitor of the alternate pathway of the complement system 37. By binding to C3b and inhibiting its interaction with factor b or by promoting the decay of existing C3b complexes, CFH inhibits C3 convertase 37. C3 protein plays a major role in complement activation and is most highly expressed in the choroid 37, 38, 39, 40. The CFH protein is a predominant cell surface- associated inhibitor of the complement in the retinal pigment epithelium-choroid complex that downregulates the alternative pathway of the complement system through inhibition of C3 activity 38, 39, 40.

Moreover, CFH binds and interacts with adrenomedullin that it is known to cause vasodilation of the choroidal vessels and to affect choroidal blood flow. In addition, it is known to increase microvascular permeability 40, 41, 42. Adrenomedullin hemodynamic activities in combination with the fact that alterations of the choroidal blood flow and hypepermeability in the choroidal vessels are the key factor in CSCR pathogenesis indicate that it might be a possible association of CFH and CSCR 40, 43.

## Complement factor H gene studies

Miki et al 43, first of all in a genetic association study, investigated the possible role of CFH in CSCR. Five single nucleotide polymorphisms (SNPs) spanning the CFH locus were genotyped in 140 CSCR patients, 934 population based controls and 335 hospital- based controls 43. They found that all 5 SNPs (rs3753394, rs800292, rs2284664, rs1329428 and rs1065489) were significantly associated with CSCR 43. The strongest association was observed with rs1329428 (Alleles(A): C/T, Odds Ratio(OR)=1.79, Confidence Intervals(CI):1.39-2.31, P=6.44x10^-6^) followed by rs1065489 (A:G/T, OR=0.59, CI:0.45-0.77, P=6.56x10^-5^), rs800292 (A:G/A, OR=1.66, CI:1.29-2.14, P=6.75x10^-5^), rs2284664 (A:C/T, OR=1.54, CI:1.19-1.98, P=7.84x10^-4)^, rs3753394 (A:T/C,OR=1.50, CI:1.16-1.94, P=0.0017) 43.Given that the 5 SNPs are correlated substantially with each other (r^2^>-0.38), Miki et al performed a conditional logistic regression analysis of these 5 SNPs which revealed that after controlling for the genetic effect of rs1329428 the remaining 4 SNPs association evidence was no longer significant (smallest Pconditional=0.477) 43. Also, after controlling for the genetic effect of rs1065489, no other single nucleotide polymorphism (SNP) showed significant association evidence (smallest Pconditional=0.174) 43. Rs1065489 showed significant evidence of association after conditioning of any SNP other than rs1329428 43. Therefore, Miki et al showed that the multiple associations within the CFH locus are not independent because of the high linkage disequilibrium across the region 43. The two strongest associated SNPs (rs1329428 and rs1065489) which are highly correlated account for the association signals detected at the locus 43. Haplotype analysis did not show stronger association than the single allele analysis 43.

Schubert et al 44 screened in total 82 SNPs from 44 genes that could be related to CSCR. In CFH gene they screened 5 SNPs (rs529825, rs3766404, rs1410996, rs1061170 and rs2284664) 44. They found significant association in rs529825 (A:A>G, OR=0.72, P=0.0161) in males samples in the joint analysis of the two used cohorts(USA and Danish cohort) 44. The joint analysis of all samples in all cohorts showed a possible association of rs529825 (A:A>G, OR=0.83, P=0.0745) and of rs2284664 (A:A>G, OR=1.22, P=0.0700) 44.

Seven SNPs (rs12144939, rs3753394, rs800292, rs1061170, rs2284664, rs1329428 and rs1065489) in CFH gene were screened by de Jong et al 45. They found after correcting for multiple testing that rs800292 (A:G/A, OR=1.50, CI:1.18-1.90, P=7.5x10^-4^) and rs1329428 (A:C/T, OR=1.47, CI:1.17-1.83, P=4.6x10^-4^) conferred an increased risk for CSCR 45. Rs 1065489 (A:G/T, OR=0.63, CI:0.45-0.87, P=0.003) was protective for CSCR 45. All of the above associations reached the significance threshold of the study 45. The rest of the tested SNPs {rs12144939 (A:G/T, OR=1.33, CI:1.03-1.70, P=0.031), rs3753394 (A:C/T, OR=0.78, CI:0.6-1.0, P=0.027), rs1061170 (A:T/C, OR=0.83, CI:0.66-1.05, P=0.065), rs2284664(A:C/T, OR=1.37, CI:1.07-1.76, P=0.009)} results showed a trend toward association but did not reached the significance threshold 45. In the same study, haplotypes were generated for rs3753394, rs800292, rs1061170, rs2284664, rs1329428 and rs1065489 45. Of the five observed CFH haplotypes, H3 (TGTCCT) was significantly protective (OR=0.54, CI: 0.32-0.91, P=0.010) for CSCR 45. The H2 haplotype (CATTTG) showed a trend towards association (OR=1.33, CI: 0.93-1.90, P=0.072) 45. Researchers noted that CFH SNPs that confer risk for CSCR were previously found to be protective for age-related macular degeneration (AMD) and vice versa 45. The same observation was also reported by Miki et al in their study of CFH SNPs 43. The effect sizes in the study of de Jong et al were slightly smaller than in the study of Miki et al which may partially explained by genetic differences that exist between the Japanese (Miki et al study) and Western European populations(de Jong et al study) 43, 45.

In a Greek origin population (41 cases and 78 controls), Moschos et al 40 investigated the potential association of five CFH SNPs (rs3753394, rs800292, rs2284664, rs1329428 and rs1065489) and CSCR susceptibility. Three out of the five tested SNPs (rs3753394, rs1329428 and rs1065489) were found to increase risk for CSCR significantly 40. Rs1329428 showed the strongest association as a risk factor for CSCR (A:G/G, OR=5.45, CI:1.83-16.29, P=0.004) followed by rs3753394 (A:T/T, OR=4.4, CI:1.31-14.89, P=0.02) and rs1065489 (A:T/T, OR=3.33, CI:1.15-9.64, P=0.04) 40. The results of rs1329428 were in line (risk alleles) with the studies of Miki et al (Japanese population) and de Jong et al (Western-European population) but the effect size in the Greek population study was higher 40, 43, 45. Also, rs3753394SNP was found as risk factor in both the studies of Moschos et al and Miki et al but showed a trend towards protection in the study of de Jong et al 40, 43, 45. Moreover, rs1065489 were found to confer risk to CSCR in opposite to previous studies that were found to be protective 40, 43, 45. Researchers interestingly noted that the rs3753394, rs1329428 and rs1065489 SNPs have an important role in CFH gene function 40.

Mohabati et al 46 analyzed potential genetic association specifically in acute CSCR patients and compare their results with known genetic associations that were identified in patients with chronic CSCR. As it concerns the CFH gene they examined six SNPs (rs800292, rs1061170, rs1065489, rs1329428, rs2284664, rs3753394) 46. Among the tested SNPs, five SNPs showed an association with acute CSCR 46. Two SNPs (rs1065489 and rs2284664) showed an association that was lost after correction for multiple testing 46. Rs800292 (A:G/A, OR=1.53, CI:1.15-2.03, P=3.06x10^-3^) and rs1329428 (A:C/T,OR=1.83, CI:1.40-2.38, P=5.87x10^-6^) were significantly associated as risk factors for acute CSCR. Rs1061170 (A: T/C, OR=0.64, CI: 0.48-0.86, P=2.82x10^-3^) was found to be significantly protective for acute CSCR 46. When compare their findings in acute CSCR patients with previous similar studies in chronic CSCR patients, they did not found significant differences among them though the effect size seemed to be larger in acute CSCR patients 46. Haplotype analysis in acute CSCR patients showed the same association that was observed in chronic CSCR patients as well but with larger effect size again 46. No genetic difference between acute and chronic CSCR patients could be found in this study but the effect size seemed to be systematically larger in acute CSCR patients, not significantly though 46.

No significant differences were found by the same group in the same SNPs of CFH gene (rs800292, rs1061170, rs1065489, rs1329428, rs2284664, rs3753394) among acute, non-severe chronic and severe chronic CSCR patients 47. Therefore researchers concluded that the three different CSCR studied phenotypes could not be differentiated based on the genetics 47.

## Genome wide association studies

Genome wide association studies are an important tool to identify potential susceptibility genes associated with complex diseases 48, 49.

Based on the fact that thickened choroid is strongly associated with the development of CSCR, Hosoda et al performed a genome wide association study (GWAS) on choroidal thickness in a Japanese community-based cohort to identify potential associated genes. They found that CFHrs800292 (P=2.05x10^-10^, Effect Allele (EF) =A) and VIPR2rs3793217 (P=6.75x10^-8^, EF=G) were significant associated with choroidal thickness. Subsequently, they tested the identified SNPs (CFHrs800292 and VIPR2rs3793217) in a case-control study to find if these SNPs are associated with CSCR. Both CFHrs800292 (A: G/A, OR=1.29, CI: 1.14-1.47, P=5.27x10^-5^) and VIPR2rs3793217 (A:A/G, OR=1.26, CI:1.11-1.43, P=4.59x10^-4^) were found to be significantly associated with the development of CSCR. For replication reasons VIPR2rs3793217 was also tested in a Korean case-control cohort where similar results (A: A/G, OR=1.21, CI:1.01-1.45, P=0.038) to the Japanese population were found 50.

A GWAS was performed on European population by Schellevis et al to identify new chronic CSCR disease loci. This GWAS identified 20 SNPs that reached genome wide significance (P<5.0x10^-8^). All of these resided at one locus on chromosome 1 in the CFH gene.Rs1329428 was the lead variant and it was associated with an increased risk for chronic CSCR (A:C/T, OR=1.57, CI:1.38-1.80, P=3.12x10^-11^). Additionally, ARFGEF1rs561753392 (A:C/T, OR=42.25, CI:9.55-186.89, P=1.33x10^-7^), PITPNC1rs76766498 (A:A/G, OR=5.25, CI:2.94-9.48, P=1,51x10^-7^), MEGF6rs118083394 (A:C/T, OR=8.36, CI:3.67-19.06, P=6.28x10^-7^), mir-29b-2/CD46rs4844392 (A:C/G, OR=0.64, CI:0.54-0.77, P=6.69x10^-7^), UpstreamGATA5rs2379120 (A:T/A, OR=0.67, CI:0.57-0.79, P=7.14x10^-7^) and RORArs541395042 (A:C/T, OR=206.11, CI:4.50-9436.68, P=7.84x10^-7^) showed suggestive association signals but they did not reach genome wide significance level. Conditional analysis of the lead SNP (CFHrs1329428) on chromosome1, that was performed, did not find any other independent signal in the CFH gene but the suggestive signal of mir-29b-2/CD46 gene was retained. Haplotype analysis was performed to further characterize the association at the CFH gene. The H2, H4 and H5 haplotypes (all contained the minor allele of rs1329428) were associated with an increased risk of chromic CSCR. The H1 and H3 haplotyes (both contained the major allele of rs1329428) were protective for CSCR. In this study, researchers using Predixcan analysis showed that potassium sodium-activated channel subfamily T member 2(KCNT2) and tumor necrosis factor receptor superfamily member 10a(TNFRSF10A) genes were expressed in a different way in patients with chronic CSCR. Also, predixcan analysis revealed changes in predicted expression of complement genes CFH, CFH related 1 (CFHR1), CFH related 4 (CFHR4) and membrane cofactor protein (MCP/CD46) between CSCR patients and control participants. Researchers concluded that their results underscore the potential importance of the complement pathway in the pathophysiology of chronic CSCR 48.

In a two stage GWAS in Japanese population rs11865049 located at SLC7A5 gene in chromosome 16q24.2 was identified to be significantly associated with CSCR (A: G/A, OR=2.10, CI: 1.62-2.67, P=9.7x10^-9^). Researchers concluded that SLC7A5 gene might be the potential candidate associated with CSCR that was not previously identified. SLC7A5 gene codes large neutral amino acid transporters small subunit1 (LAT1). In the eye LAT1 is expressed in retinal vascular endothelial cells, Müller cells, ciliary non pigmented epithelium and in the RPE. LAT1 is considered to have a crucial role in transportation of various neutral amino acids at the basolateral plasma membrane. Also, it can exchange intracellular glutamine for external large neutral amino acids. Abnormality in the transporting system associated with LAT1 may be involved in the accumulation of subretinal fluid that it is noticed in CSCR. Additionally, in this study researchers examined the association of CFH variants with CSCR. Though they did not reach genome wide significance level (P<5x10^-8^) rs1329428 (OR=1.432, P=1.73x10^-5^) and rs800292 (OR=1.484, P=1.98x10^-6^) of CFH gene showed an association with CSCR 49.

To investigate the role of rare (protein-altering) variants in chronic CSCR, Shellevis et al (2019) performed exome sequencing in a case control-cohort (269 cases and 1586 controls). They identified no significant associations in both the entire cohort and the male cohort. Gene- based analyses found four chronic CSCR-associated genes in the female cohort. Significant associations were observed with the PIG2 (P_SKAT_=9.19X10^-7^ and P_SKAT-O_=2.48X10^-6^), RSAD1 (P_SKAT_=1.92X10^-7^ and P_SKAT-O_=8.57X10^-8^), DUOX1 (P_SKAT_=1.03X10^-6^), and LAMB3 (P_Burden_=1.40X10^-6^ and P_SKAT-O_=1.14X10^-6^) genes in female chronic CSCR patients. This difference was statistical significant (OR=3.67, CI: 2.09-6.46, P=1.92x10^-6^). Researchers stated that the sex specific associations that were identified in their study points to a possible interaction between sex and genetics for chronic CSCR 51.

A large-scale GWAS for CSCR was performed by Hosoda et al (2019). This GWAS was followed by replication analyses using three independent Japanese and European cohorts. In the first stage (discovery GWAS), researchers found that both rs13278062 at TNFRSF10A-LOC389641 (OR=1.38, CI: 1.22-1.57, P=5.94x10^-7^) and rs6061548 near GATA5 (OR=1.64, CI: 1.36-1.98, P=2.52x10^-7^) exceeded the suggestive threshold of P-value (P-value=1.0x10^-6^). In the first replication stage using 278 CSCR Japanese samples both rs13278062 at TNFRSF10A-LOC389641 (OR=1.35, CI: 1.13-1.60, P=8.97x10^-4^) and rs6061548 near GATA5 (OR=1.39, CI: 1.07-1.80, P=1.28x10^-2^) found to be significantly associated with CSCR. Second replication analysis in another Japanese CSCR case-control cohort (137cases and 1153controls) showed that rs6061548 near GATA5 was also significantly associated with CSCR (OR=2.29, CI: 1.60-3.27, P=5.55x10^-6^) but TNFRSF10A-LOC389641rs13278062 showed a trend towards an association (OR=1.19, CI: 0.92-1.53, P=0.189). The third replication analysis in a European case-control cohort (521 cases and 3577 controls) showed that both TNFRSF10A-LOC389641rs13278062 (OR=1.36, CI: 1.18-1.56, P=1.47x10^-5^) and rs6061548 near GATA5 (OR=1.60, CI: 1.23-2.07, P=5.80x10^-4^) were significantly associated with the development of CSCR. Meta-analysis of all data showed robust association of both SNPs with the development of CSCR (OR=1.35, CI: 1.24-1.46, P=1.26x10^-13^ for TNFRSF10A-LOC389641rs13278062 and OR=1.63, CI: 1.44-1.85, P=5.36x10^-15^ for rs6061548 near GATA5). Researchers searched both the Eyeintegration database (https://eyeintegration.nei.nih.gov/, v1.01) and the Ocular Tissue database (https://genome.uiowa.edu/otdb/) where they found that in both databases the expression of TNFRSF10A and GATA5 was stronger in the adult human RPE/choroid than in the adult human retina. Researchers stated that TNFRSF10A may confer risk to develop CSCR by modulating hormone secretion from the adrenal glands and GATA5 may contribute risk to CSCR through vascular endothelial dysfunction in the choriocapillaries. In this study, researchers also tested two previously reported susceptibility loci for CSCR, CFHrs1329428 and SLC7A5rs11865049. CFHrs1329428 showed a significant association with CSCR (Effect allele=T, OR=1.17, CI: 1.03-1.32, P=1.45x10^-2^) but SLC7A5rs11865049 did not reach a significant value (Effect allele=A, OR=1.15, CI: 0.91-1.44, P=0.24). Two new susceptibility loci for CSCR (TNFRSF10A-LOC389641rs13278062 and rs6061548 near GATA5) were found in this study by using a large number of cases and controls (1546 cases and 13029 controls in total) 52.

## Other genes studies

It has been noticed that CSCR patients have increased levels of plasminogen activator inhibitor type-1 (PAI-1) 53, 54. Platelet aggregation induced by elevated PAI-1 level results in arterial filling with capillary and venous dilation due to microthrombus formation 55. Furthermore tissue-type plasminogen activator(t-PA), which is the main activator of fibrinolysis and it is directly blocked by PAI-1, was found to be significantly lower in CSCR patients^4^, ^55^. PAI-1 plasma concentrations are affected by the PAI-1 promoter insertion/deletion of guanosine (4G/5G) polymorphism 53, 56. Higher PAI-1 levels were found among homozygotes of the PAI-1 4G allele compared to heterozygotes and homozygotes of the PAI-1 5G allele 57.

A potential association between the 4G/5G polymorphism of PAI-1 gene in patients with acute CSCR has been studied by Sari et al in 60 patients and 50 controls. In this study, no significant difference (P=0.70) was found in the distribution of genotypes between CSCR patients and control subjects. The CSCR patients had a significantly higher serum PAI-1 level that the control subjects (P=0,001). In both groups no association was seen between the serum PAI-1 concentrations and 4G/5G genotype. According to their results, researchers stated that 4G/5G polymorphism in the promoter of PAI-1 gene is not a risk factor for the elevated serum PAI-1 concentrations and consequent development of CSCR 53.

In a bigger cohort (172 CSCR patients and 313 control subjects), Male et al tested both a gene polymorphism characterized by a C to T transition at position -7351 of the t-PA gene (t-PA -7351 C>T) and PAI-1 gene 4G/5G polymorphism to find a potential association between these and CSCR. No statistical significant differences were found in both tested genes. After adjusting for age and gender, researchers found that homozygosity for both tested gene variants was not significantly associated with the development of CSCR (PAI-1 4G/4G: OR=1.21, CI: 0.77-1.92, P=0.41 and t-PA -7351CC: OR=0.91, CI: 0.62-1.33, P=0.62). This study confirmed the findings of the Sari et al that also investigated the role of the PAI-1 4G/5G polymorphisms 57.

De Jong et al investigated the potential association of 19 known AMD-associated loci with chronic CSCR. ARMS2rs10490924, CFHrs12144939, C2-CFBrs429608, C3rs2230199, TIMP3rs9621532, APOErs4420638, CETPrs3764261, VEGFArs943080, TNFRSF10Ars13278062, LIPCrs493258, CFIrs10033900, COL10A1rs3812111, COL8A1-FILIP1Lrs13081855, IER3-DDR1rs3130783, SLC16A8rs8135665, TGFBR1rs334353, RAD51Brs8017304, ADAMTS9rs6795735 and B3GALTLrs9542236 SNPs were tested in a case-control cohort. After correcting for multiple testing, only ARMS2rs10490924 was found to be significantly associated as a protective factor for chronic CSCR (A: G/T, OR=0.64, CI: 0.49-0.85, P=0.002). ARMS2 was shown to interact with components of the extracellular matrix 45, 58. It is interested that the association of ARMS2rs10490924 with chronic CSCR is protective whereas it confers risk to AMD (OR=2.76 for AMD and OR=0.64 for chronic CSCR).CFHrs12144939(A:G/T, OR=1.33, CI:1.03-1.70, P=0.031), TNFRSF10A(A:T/G, OR=0.73, CI:0.59-0.90, P=0.004) and ADAMTS9rs6795735 (A:C/T, OR=1.25, CI:1.01-1.54, P=0.047) showed a trend toward association with chronic CSCR but did not reach the significance level(P<0.0026)^45^.

Based on their function in pathways implicated in CSCR (mainly stress response, steroid metabolism and choroidal/endothelial permeability) 82 SNPs from 44 genes were screened by Schubert et al. Initial screening was performed on a US cohort of CSCR patients and healthy controls. SNPs with suggestive association from the US cohort were tested as well in a replication Danish cohort of patients and controls. CFHrs2298877, MAPK1rs1063311, CFHrs529825 and CFHrs2284664 SNPs were marginally significant in one of the two cohorts and in the joint analysis which could mean a no significant finding clinically. However, four out of ten SNPs from the CDH5 gene were independently associated significantly with CSCR development in the US and Danish cohort and were also associated in the joint analysis of the male subgroup from both cohorts {Rs7499886 (A:A>G, OR=1.47, CI:1.2-1.8, P=0.00012), rs1073584 (A:C>T, OR=0.70, CI:0.57-0.87,P=0.0014), rs1130844 (A:C>T, OR=0.78, P=0.0024) and rs2344564 (A:C>T, OR=1.43, P=0.012)}. No significant differences were observed (P>0.05) between the chronic and acute forms of CSCR. CDH5 gene is an essential gene for vascular homeostasis. Researchers proposed that CDH5 gene variants in combination with triggering events, such as corticosteroid treatment or severe hormonal imbalance, could be responsible for a substantial proportion of CSCR in the male population 44.

Serum glucocorticoid kinase gene-1(SGK-1), which is a signaling molecule downstream of glucocorticoid receptors, was investigated for association with chronic CSCR. Researchers did not find any association of rs1057293 (P=0.68) and rs1743966 (P=0.28) polymorphisms of SGK-1 gene using a case-control cohort (32 patients and 32 controls). Despite this, they identified a new polymorphism M32V in two people in the patient group 59.

Because complement component 4 gene (C4) plays an important role in the complement system and it is also associated with the hypothalamic-pituitary-adrenal axis, Breukink et al assessed the copy number variations of the C4 gene in a chronic CSCR cohort (197 patients and 303 controls). No significant difference was found for C4A genomic copy number (range: 0-6, P=0.649) between chronic CSCR patients and healthy controls. However, C4B distribution was significantly different between patients and controls (range: 0-4, P=0.0018). In general, chronic CSCR patients found to carry lower C4B copy numbers than control subjects. Carrying no copies of C4B was significantly associated with an increased risk to develop chronic CSCR (OR=2.61, CI: 1.05-6.52, P=0.039). Carrying one copy of C4B showed a trend towards positive association to chronic CSCR development but the results were not significant (OR=1.47, CI: 0.96-2.26, P=0.080). Carrying three copies of C4B was found to be significantly protective to develop chronic CSCR (OR=0.45, CI: 0.24-0.85, P=0.014) whereas carrying four copies of C4B was not found to be significantly associated with chronic CSCR (OR=0.809, CI: 0.145-4.503, P=0.808). The total C4 genomic copy number between patients and controls was not found to be different (P=0.148). Researchers stated that their findings make stronger the hypothesis of a possible involvement of the complement system in the chronic CSCR aetiopatholgy 60.

Due to its potential protective role against oxidative stress in the retina, glutathione S-transferase MU-1 (GSTM1) gene polymorphisms was investigated, in a well-defined Greek cohort (41 cases and 78 controls), for possible association with CSCR. No association was found between GSTM1 gene polymorphisms and CSCR patients (GSTM1 null genotype OR=0.51, CI: 0.22-1.21, P=0.15) and therefore the hypothesis that oxidative stress may contribute to the pathogenesis of CSCR was not confirmed in this study 40.

The glucocorticoid receptor (GR) and the mineralocorticoid receptor (MR) has been suggested to be involved in the CSCR pathogenesis^4^, ^61^.The GR is encoded by the NR3C1 gene and the MR is encoded by the NR3C2 gene. Van Dijk et al investigated in a case-control cohort (336 cases and 1314 controls) if genetic variants in NR3C1 (rs56149945, rs41423247 and rs6198) and NR3C2 (rs2070951 and rs5522) are associated with chronic CSCR. Also, using a haplotype analysis they investigated the effect of the two variants in NR3C2. Genetic variants in the NR3C1 gene (rs56149945, rs41423247 and rs6198) were not found to be significantly associated with chronic CSCR. No association was also found for rs5522 of NR3C2 gene with chronic CSCR. On the other hand, rs2070951 of NR3C2 gene polymorphism was found to be significantly associated as a risk factor for the development of chronic CSCR (A: C/G, OR=1.29, CI: 1.08-1.53, P=0.004). Haplotype analysis showed that haplotype CA (C for rs2070951 and A for rs5522) was protective for the development of chronic CSCR (OR=0.72, CI: 0.60-0.87, P<0.001) and haplotype GA (G for rs2070951 and A for rs5522) increased the risk for chronic CSCR (OR=1.39, CI: 1.15-1.68, P=0.004). Researchers stated that their results indicate a possible role for the mineralocorticoid receptor in chronic CSCR pathogenesis but further studies with larger cohorts are needed to exclude the involvement of the four gene polymorphisms (rs56149945, rs41423247, rs6198 of NR3C1 gene and rs5522 of NR3C2 gene) that were not associated with chronic CSCR in their study 61.

Mohabati et al, apart from SNPs of CFH gene, assessed whether SNPs in ARMS2 gene (rs10490924), NR3C2 gene (rs2070951) and the copy numbers of C4B gene are associated with acute CSCR in a white patient cohort. Also, they compared their results with the results of previous studies in chronic CSCR patients to assess whether there are significant differences between acute and chronic CSCR patients. No association was found with the rs10490924 SNP in ARMS2 gene in acute CSCR patients (A: G/T, OR=0.76, CI: 0.54-1.07, P=0.111). Rs2070951 of NR3C2 gene SNP showed an initial significant association that was lost after correction for multiple testing (A: C/G, OR=1.33, CI: 1.03-1.71, P=0.0287). C4B gene copies distribution was significantly different between acute CSCR patients and controls (P=0.0042). Carriers of 3 C4B copies found to have a reduced risk of developing acute CSCR (OR=0.27, CI: 0.12-0.63, P=0.002). No significant differences were found in the tested SNPs between acute and chronic CSCR patients 46.

In another study by the same group, no significant differences in rs10490924 of ARMS2 gene, rs2070951 of NR3C2 gene and C4B gene copy numbers were found among the three different studied CSCR phenotypes (Acute, non-severe chronic and severe chronic). Researchers concluded that there is an overlap in the genetic predisposition of different CSCR phenotypes 47.

## Conclusion

Central serous chorioretinopathy (CSCR) is a disorder of unknown etiology that is characterized by neurosensory detachment of the central retina secondary to fluid leakage through the retinal pigment epithelium. It has an incidence of 9.9 per 100000 in men and 1.7 per 100000 in women. CSCR affects predominantly middle aged people and it is, usually, a self-limiting disorder but it occasionally persists and may cause visual impairment due to permanent damage to retinal pigment epithelium and photoreceptors.

Pathophysiology of CSCR is not well understood but choroid, RPE and hormonal pathways seem to play important roles. Reports of familial CSCR indicated that there is a genetic background for the disease. Several candidate gene studies were performed to reveal genetic associations of CSCR. CFH gene genetic variants have been found to be associated with CSCR in numerous studies. Populations with different ethnic origin showed similar associations of CFH gene polymorphisms with CSCR. CFH gene has an important role in the alternate pathway of the complement system. Other gene associations that have been found in studies were between CSCR and SNPs in ARMS2, CDH5, NR3C2, TNFRS10A, VIPR2, GATA5, PIGZ, DUOX1, RSAD1, LAMB3, SLC7A5 and copy number variations of the C4B gene. Interestingly, genetic studies showed that genetic variants that are protective for AMD confer risk to CSCR and vice versa. Genetic studies in different CSCR phenotypes did not find significant differences among them and therefore it seems that there is a common genetic basis between different CSCR phenotypes.

The genetic background of CSCR has started to be revealed but more genetic studies are needed to have a clearer picture on CSCR genetics. By increasing our knowledge on CSCR genetics and by understanding better the pathophysiology of the disease, better and more personalized diagnostic and therapeutic approaches to CSCR could be developed.

## Abbreviations

CSCR: Central Serous Chorioretinopathy
RPE: Retinal Pigment Epithelium
OCT: Optical Coherence Tomography
FA: Fluorescein Angiography
ICGA: Indocyanine Green Angiography
EDI-OCT: Enhanced Depth Imaging Optical Coherence Tomography
CFH: Complement Factor H
SNPs: Single Nucleotide Polymorphisms
A: Alleles
OR: Odds Ratio
CI: Confidence Interval
SNP: Single Nucleotide Polymorphism
AMD: Age-related Macular Degeneration
GWAS: Genome Wide association Study
EF: Effect Allele
LAT1: Large neutral amino acid transporters small subunit 1
PAI-1: Plasminogen Activator Inhibitor Type-1
t-PA: Tissue-type Plasminogen Activator
GR: Glucocorticoid Receptor
MR: Mineralcorticoid Receptor
